# Comparison of intraocular pressure measured by ocular response analyzer and Goldmann applanation tonometer after corneal refractive surgery: a systematic review and meta-analysis

**DOI:** 10.1186/s12886-019-1288-6

**Published:** 2020-01-10

**Authors:** Hui Zhang, Zhengtao Sun, Lin Li, Ran Sun, Haixia Zhang

**Affiliations:** 1 School of Biomedical Engineering, Capital Medical University, Beijing, 100069 China; 2 Beijing Key Laboratory of Fundamental Research on Biomechanics in Clinical Application, Capital Medical University, Beijing, 100069 China; 3 Department of Ophthalmology, Xuanwu Hospital, Capital Medical University, Beijing, 100053 China; 4 School of Engineering, University of Liverpool, Liverpool, UK

**Keywords:** Corneal refractive surgery, Intraocular pressure, Ocular response analyzer, Goldmann applanation tonometer, Meta-analysis

## Abstract

**Background:**

Accurate measurement of intraocular pressure (IOP) after corneal refractive surgery is of great significance to clinic, and comparisons among various IOP measuring instruments are not rare, but there is a lack of unified analysis. Although Goldmann Applanation Tonometer (GAT) is currently the internationally recognized gold standard for IOP measurement, its results are severely affected by central corneal thickness (CCT). Ocular Response Analyzer (ORA) takes certain biomechanical properties of cornea into account and is supposed to be less dependent of CCT. In this study, we conducted the meta-analysis to systematically assess the differences and similarities of IOP values measured by ORA and GAT in patients after corneal refractive surgery from the perspective of evidence-based medicine.

**Methods:**

The authors searched electronic databases (MEDLINE, EMBASE, Web of science, Cochrane library and Chinese electronic databases of CNKI and Wanfang) from Jan. 2005 to Jan. 2019, studies describing IOP comparisons measured by GAT and ORA after corneal refractive surgery were included. Quality assessment, subgroup analysis, meta-regression analysis and publication bias analysis were applied in succession.

**Results:**

Among the 273 literatures initially retrieved, 8 literatures (13 groups of data) with a total of 724 eyes were included in the meta-analysis, and all of which were English literatures. In the pooled analysis, the weighted mean difference (WMD) between IOPcc and IOP_GAT_ was 2.67 mmHg (95% CI: 2.20~3.14 mmHg, *p* < 0.0001), the WMD between IOPg and IOP_GAT_ was − 0.27 mmHg (95% CI: − 0.70~0.16 mmHg, *p =* 0.2174). In the subgroup analysis of postoperative IOPcc and IOP_GAT_, the heterogeneity among the data on surgical procedure was zero, while the heterogeneity of other subgroups was still more than 50%. The comparison of the mean difference of pre- and post-operative IOP (∆IOP) was: mean-∆IOPg > mean-∆IOP_GAT_ > mean-∆IOPcc.

**Conclusions:**

IOPcc, which is less dependent on CCT, may be more close to the true IOP after corneal refractive surgery compared with IOPg and IOP_GAT_, and the recovery of IOPcc after corneal surface refractive surgery may be more stable than that after lamellar refractive surgery.

## Introduction

Corneal refractive surgery has become an extremely popular procedure to correct ametropia, such as myopia and hyperopia [1]. Corneal refractive surgery is mainly divided into surface refractive surgery and lamellar refractive surgery. The former mainly includes Photorefractive Keratectomy (PRK), Laser-assisted Subepithelial Keratomileusis (LASEK) and Epipolis Laser in Situ Keratomileusis (EPI-LASIK), while Laser-assisted in Situ Keratomileusis (LASIK) and Femtosecond Laser-assisted LASIK (FS-LASIK) belong to the latter [2].

No matter what kind of refractive surgery the patient had undergone, their central corneal thicknesses (CCT) decreased, and corneal thickness affected the measured intraocular pressure (IOP) [3]. Therefore, the accurate measurement of IOP after refractive surgery is one of the most challenging problems. In addition, there are risks of steroid-induced glaucoma and secondary keratoconus after corneal refractive surgery, so it is of great clinical significance to accurately measure postoperative IOP for the diagnosis and treatment of ophthalmology [4].

At present, there are several devices for measuring IOP, such as Goldmann applanation tonometer (GAT), noncontact tonometer (NCT), iCare rebound tonometer (iCare RBT) and Ocular Response Analyzer (ORA) etc. [5] Among them, the GAT (Haag Streit, Könitz, Switzerland) is regarded as the gold standard for IOP measurement [6]. It follows the Imbert-Fick principle which is based on the relationship between IOP, the outlet force, and the applanation area to measure IOP in contact. But when Goldmann [7] introduced this tonometer to measure IOP, he clearly pointed out the defects of his equipment, which measurement value (IOP_GAT_) was inevitably affected by CCT. And then some researchers also proposed that GAT could provide a correct value for the IOP when the corneal thickness was about 520 μm [8], its accuracy would gradually deteriorate when the corneal thickness deviated from this size. Therefore, the IOP_GAT_ is known to be significantly affected by CCT [9]. ORA (Reichert, Depew, NY, USA) is a kind of noncontact tonometer. During the measurement process, the amplitude of the air pulse pressure at the corneal apex change over time, and the corneal movement is in response to increased and decreased pressure amplitude. Two air pulse pressure values (P1 and P2) are recorded at the inward and outward applanation events. Due to the indentation of the cornea by an air-puff causing a dynamic time-dependent response, ORA can provide extra information about IOP and corneal biomechanics. In the output parameters of ORA, Goldmann-correlated IOP (IOPg) is the mean of these applanation pressures (IOPg = (P1 + P2)/2). Corneal hysteresis (CH) is the difference in applanation pressures (CH = C × (P1 − P2)) and is an indication of viscous damping in the cornea. The corneal resistance factor (CRF) captures the overall viscoelastic behavior of the cornea [10]. The correction of IOP according to CH could reduce the measurement of IOP by corneal factors, that is, Corneal-Compensated Intraocular Pressure (IOPcc) [11]. IOPcc is claimed to measure IOP independent of CCT and takes certain biomechanical properties of cornea into account.

Many studies have been done on comparing the IOP measured by the GAT and ORA after the corneal refractive surgery. But each study is just for one or two types of refractive surgery, so the conclusions of each study are lack of integrity. Therefore, in this work, we gave a systematic review and meta-analysis on the three types of IOP (IOPcc, IOPg, IOP_GAT_) measured by ORA and GAT after corneal refractive surgery, and hoped to draw a more comprehensive conclusion on IOP of post operation.

## Methods

### Search strategy

We searched foreign language electronic databases of MEDLINE, EMBASE, Web of science, Cochrane library and Chinese electronic databases of CNKI and Wanfang. The search terms used were “ocular response analyzer” or “ORA”, “Goldmann applanation tonometer” or “GAT”, “intraocular pressure” or “IOP”. The publication period was from Jan. 2005 to Jan. 2019, and references to all of the retrieved literature were supplemented.

Two investigators (HZ and ZS) independently searched the studies, screened identified abstracts and articles in duplicate, extracted the available data from eligible studies, and assessed the study quality.

### Inclusion and exclusion criteria

Studies describing IOP comparisons measured by GAT and ORA in their title or abstract were retrieved for full text review. Inclusions for analysis were restricted to: 1) study participants underwent corneal refractive surgery and IOP was measured with ORA and GAT after surgery; 2) mean and standard deviation of three IOP measurements (IOPcc, IOPg and IOP_GAT_) could be extracted from studies. Exclusion criteria applied were as follows: 1) studies done before 2005; 2) reviews or animal studies; 3) studies with no definite follow-up time; 4) studies comparing IOP with other conditions such as glaucoma, keratoconus, diabetes; 5) studies reported by other language (non-Chinese, non-English).

### Data extraction and quality assessment

The following available data were extracted from eligible studies: the name of first author and the year of publication (name/y), country in which the study was carried out (country), study design (retrospective or prospective), the number of eyes included in the study (sample size), mean and standard deviation of age (mean age ± SD), surgical method, surgical procedure (lamellar corneal refraction surgery or surface corneal refractive surgery), postoperative follow-up time (post-op follow-up), and mean and standard deviation of IOP measurements (IOPcc, IOPg and IOP_GAT_) after corneal refractive surgery. Any differences in data abstraction were resolved by consensus and discussion with the other authors.

The study quality was assessed by using the Quality Assessment for Diagnostic Accuracy Studies 2 (QUADAS2) checklist [12]. The patient selection risk of bias question 2 (“Was a case-control design avoided?”), and index test risk of bias question 2 (“If a threshold was used, was it prespecified?”) were excluded from the checklist because they did not apply to the current review [13].

The effect was expressed by weighted mean difference (WMD) and 95% confidence interval (CI). The heterogeneity test was performed by chi-square test, and heterogeneity index (*I*^*2*^) was used to assess heterogeneity quantitatively. If *p* ≥ 0.05 and *I*^*2*^ < 50%, multiple sets of data were considered to be homogeneous, and fixed effect model was selected for calculation and combined effect quantity. On the contrary, it was considered that there was heterogeneity, and random effect model was selected for correction [14]. Publication bias was assessed via Egger precision-weighted linear regression. We also performed subgroup analysis and univariate meta-regression to explain possible sources of statistical heterogeneity when there were differences. The prespecified subgroups of interest were study design (retrospective compared with prospective), surgical procedure (lamellar corneal refractive surgery compared with surface corneal refractive surgery), and post-op follow-up (time ≤ 1 month compared with time > 1 month, time ≤ 3 months compared with time > 3 months). And meta-regression was also performed for these three study characteristics respectively. The quality assessment process was completed by Review Manager (version 5.3), and the rest of the analysis was performed by R programming language software (version 3.5.2).

## Results

### Search results

**Fig. 1:**
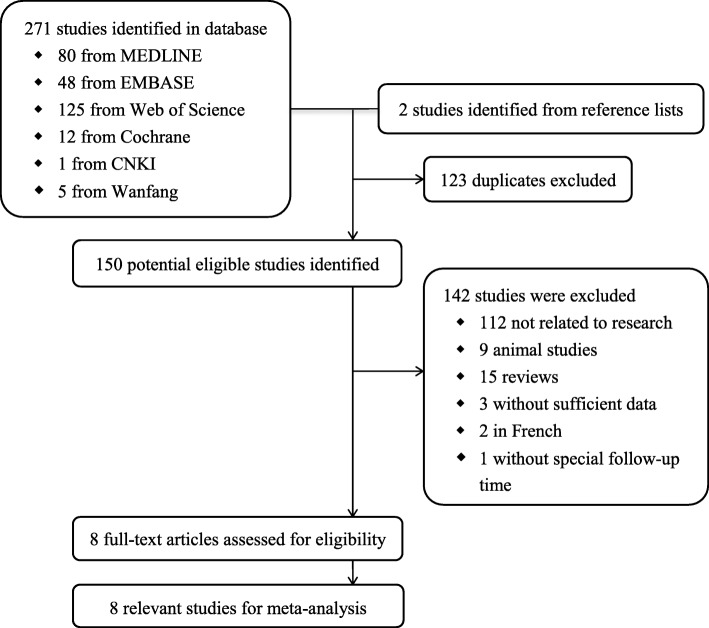
Flow chart of study identification, exclusion, and inclusion in the meta-analysis Statistical analysis

The method used to select the studies is shown in Fig. 1. The initial search identified 273 studies (80 from MEDLINE, 48 from EMBASE, 125 from Web of Science, 12 from Cochrane, 1 from CNKI, 5 from Wanfang and 2 studies identified from reference lists). After removing duplicates, 150 citations were reviewed. A total of 142 publications were excluded for the following reasons: 112 did not belong to the comparison for IOP after corneal refractive surgery, 9 were conducted with animals, 15 were reviews, 3 were unable to extract the mean or standard deviation of the three kinds of IOP, 2 studies were in French without an English translation, and 1 without special follow-up time. Therefore, the meta-analysis was comprised of 8 full articles.

### Study characteristics

The characteristics of the studies included in our analysis are presented in Table 1. Of the 8 articles, Kirwan’s, Qazi’s and Denise’s all included data of different follow-up time, so there were 13 groups of data in this study. Five of the 8 studies included were prospective and 3 were retrospective. Three of the studies were conducted in America and two studies were conducted in China, others were each in Ireland, Korea, and Iran. The sample sizes varied from 28 to 148, and the total was 724. The average age was between the 20 and 40 years old. The longest follow-up time was 12 months, the shortest was only 1 week, and the rest were 1 month, 3 months and 6 months respectively.

**Table 1:** Characteristics of studies included in the meta-analysis

Name/y	Country	Retrospective or prospective	Sample size	Mean age ± SD (years)	IOP(mmHg)			Surgical method	Lamellar or surface	Follow-up time
					IOPcc	IOPg	IOP_GAT_			
Kirwan_a/2008 [15]	Ireland	Prospective	90	35.6 ± 9.3	13.1 ± 1.9	10.2 ± 2.1	9.6 ± 1.7	LASIK	Lamellar	3 months
Kirwan_b/2008 [15]	Ireland	Prospective	35	37.3 ± 11.7	13.8 ± 2.7	10.7 ± 2.5	11.0 ± 2.1	LASEK	surface	3 months
Fan/2011 [16]	China	Retrospective	148	22.9 ± 4.64	13.91 ± 2.26	9.79 ± 2.52	10.09 ± 2.43	LASIK	Lamellar	6 months
Hong/2015 [17]	China	Retrospective	50	21.8 ± 5.9	15.3 ± 2.4	12.5 ± 2.1	13.0 ± 2.3	LASIK	Lamellar	3 months
Pepose/2007 [18]	America	Prospective	66	39.6 ± 11.4	13.1 ± 2.0	10.6 ± 2.6	12.0 ± 2.7	LASIK	Lamellar	1 week
Qazi_a/2009 [19]	America	Prospective	28	39.0 ± 12.0	13.37 ± 2.53	11.29 ± 3.08	11.53 ± 2.45	LASIK	Lamellar	6 months
Qazi_b/2009 [19]	America	Prospective	30	41.0 ± 9.0	14.24 ± 2.82	10.07 ± 3.55	11.86 ± 2.74	LASEK	surface	6 months
Denise_a/2011 [20]	America	Prospective	51	36.0 ± 8.0	16.40 ± 2.43	13.16 ± 3.08	13.82 ± 2.56	EPI-LASIK	surface	1 month
Denise_b/2011 [20]	America	Prospective	51	36.0 ± 8.0	16.00 ± 2.60	13.09 ± 3.25	13.34 ± 2.58	EPI-LASIK	surface	3 months
Denise_c/2011 [20]	America	Prospective	51	36.0 ± 8.0	14.66 ± 2.30	11.73 ± 2.56	11.86 ± 2.71	EPI-LASIK	surface	6 months
Denise_d/2011 [20]	America	Prospective	51	36.0 ± 8.0	15.09 ± 2.30	12.11 ± 2.54	12.40 ± 2.67	EPI-LASIK	surface	12 months
Shin/2015 [21]	Korea	Retrospective	40	26.25 ± 7.23	13.64 ± 2.09	10.27 ± 2.26	10.83 ± 2.83	FS-LASIK	Lamellar	1 month
Zare/2012 [22]	Iran	Prospective	33	26.9 ± 5.0	15.25 ± 3.24	14.15 ± 2.73	12.42 ± 2.14	PRK	surface	3 months

*LASIK* Laser-assisted in Situ Keratomileusis, *LASEK* Laser-assisted Subepithelial Keratomileusis, *EPI-LASIK* Epipolis Laser in Situ Keratomileusis, *FS-LASIK* Femtosecond Laser-assisted LASIK, *PRK* Photorefractive Keratectomy

### Quality assessment

**Fig. 2:**
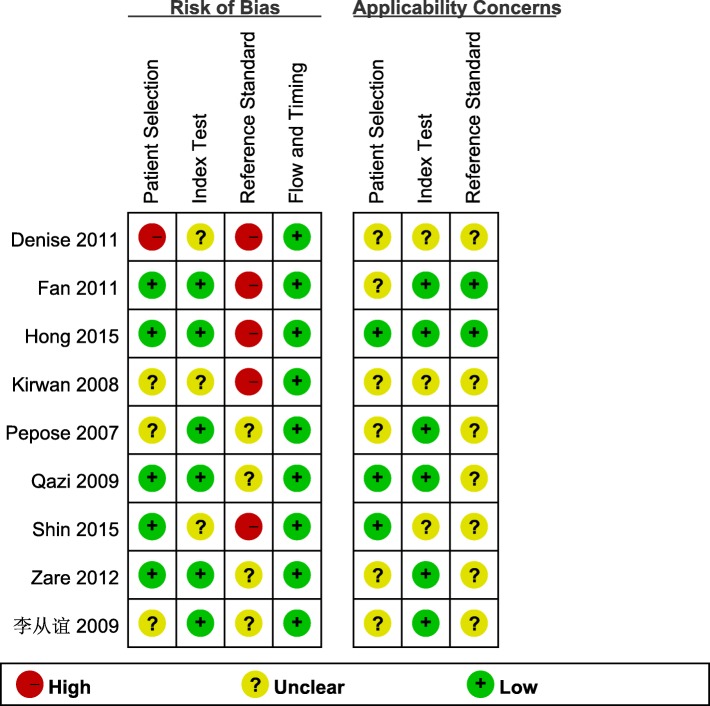
Quality assessment of studies included in the meta-analysis

The QUADAS2 tool was applied to assess for bias and the quality evaluation results of the included literatures are shown in Fig. 2. The reference standard in five articles was highly biased [15–17, 20, 21] and the patient selection in only one article was highly biased [20]. For flow and timing, all literatures showed lowly biased [15–22]. In general, the quality of the included literatures was relatively high.

### Analysis of postoperative IOPcc and IOP_GAT_

**Fig. 3:**
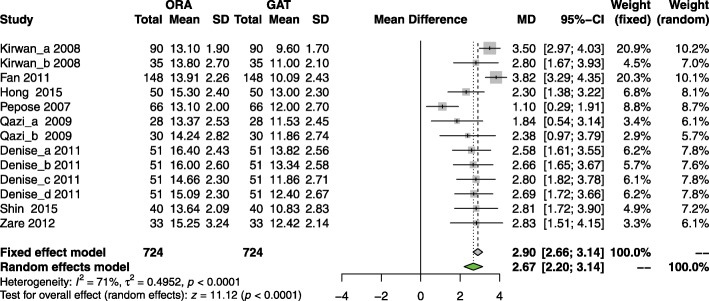
Forest plot of the correlation between postoperative IOPcc and IOP_GAT_

Figure 3 shows the forest plot of the correlation between postoperative IOPcc and IOP_GAT_. There was significant heterogeneity among the groups of data (*p* < 0.0001, *I*^*2*^ = 71%), so the random effect model was used for analysis. In the pooled analysis, the WMD between IOPcc and IOP_GAT_ was 2.67 mmHg (95% CI: 2.20~3.14 mmHg, *p* < 0.0001, Fig. 3). The Egger statistic (*p* = 0.028) revealed there was certain publication bias.

We performed subgroup analysis using the study design, surgical procedure and postoperative follow-up time as sub-group criteria respectively. In the subgroup analysis of postoperative IOPcc and IOP_GAT_, as shown in Table 2, the heterogeneity among the data on surgical procedure was zero, while the heterogeneity of other subgroups was still more than 50%.

**Table 2:** Analysis results for each subgroup of IOPcc and IOP_GAT_

Subgroup factor	Group standard	Number of data group	Q-value	*P*-value	*I*^*2*^	95% CI
Study design	retrospective	3	8.90	0.0117	77.5%	3.04 (2.02~4.05)
	prospective	10	25.86	0.0022	65.2%	2.54 (2.02~3.07)
Surgical procedure	lamellar	6	38.97	<0.0001	87.2%	2.61 (1.73~3.50)
	surface	7	0.37	0.9991	0%	2.69 (2.28~3.09)
Post-op follow-up	> 1 month	10	18.85	0.0265	52.3%	2.90 (2.48~3.32)
	≤1 month	3	8.23	0.0163	75.7%	2.12 (1.01~3.24)
	> 3 months	5	12.36	0.0149	68.0%	2.83 (2.07~3.60)
	≤3 months	8	24.72	0.0009	72.0%	2.57 (1.96~3.18)
Total		13	40.92	<0.0001	71.0%	2.67 (2.20~3.14)

The meta-regression results showed no statistical significance for the effect of three characteristics (study design, surgical procedure, post-op follow-up) on heterogeneity, namely, study design (*p* = 0.9747), surgical procedure (*p* = 0.0976), post-op follow-up (*p* = 0.2983 (1 month), *p* = 0.5096 (3 months)).

### Analysis of postoperative IOPg and IOP_GAT_

**Fig. 4:**
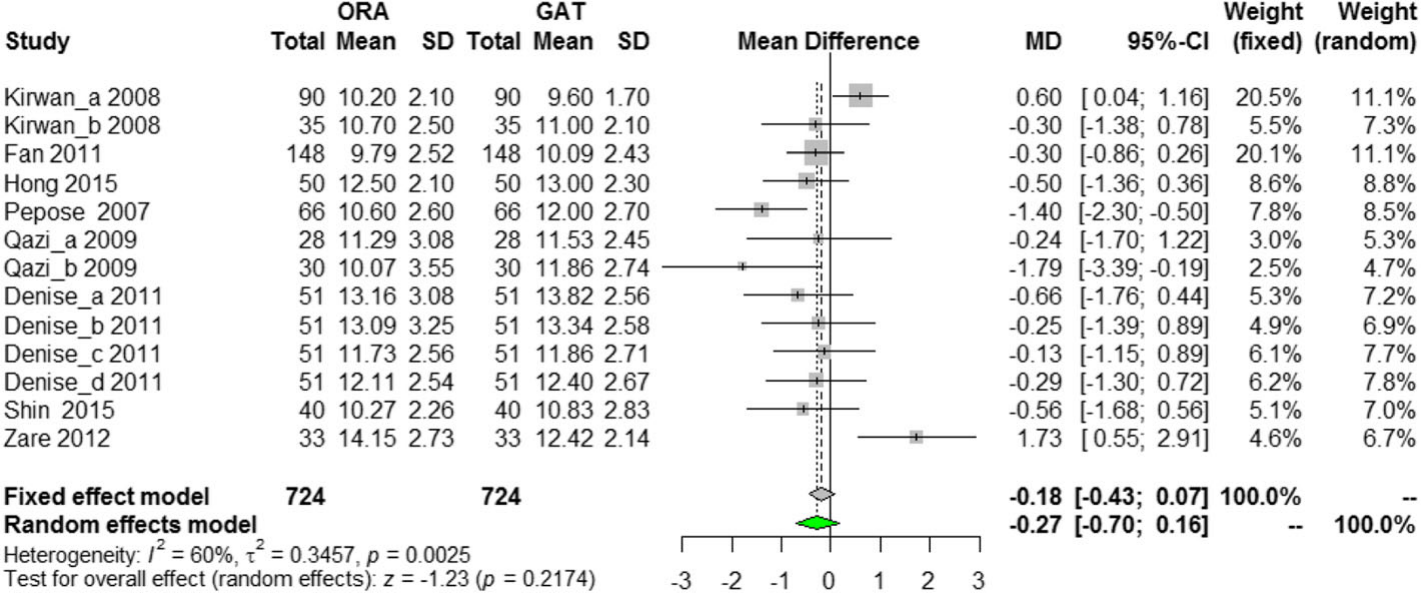
Forest plot of the correlation between postoperative IOPg and IOP_GAT_

Figure 4 shows the forest plot of the correlation between postoperative IOPg and IOP_GAT_. There also was little heterogeneity among the data of groups (*p* = 0.0025, *I*^*2*^ = 60%), and random effect model was used for analysis. In the pooled analysis, the WMD between IOPg and IOP_GAT_ was − 0.27 mmHg (95% CI: − 0.70~0.16 mmHg, *p =* 0.2174, Fig. 4) and Egger statistics (*p* = 0.1339) showed no publication bias.

Although the Fig. 4 showed that there was some heterogeneity between IOPg and IOP_GAT_ after operation, there was no significant difference between them in general, so the heterogeneity between IOPg and IOP_GAT_ had not been analyzed.

### Comparison of IOP pre- and post-operative surgery

In the 8 studies, only four groups of data [18–21] contain preoperative IOP and ∆IOP, where ∆IOP refers to the difference between the value IOP obtained from pre- and post-operation. The data were summarized in Table 3. Through the meta-analysis, the WMD between preoperative IOPcc and IOP_GAT_ was 1.52 mmHg (95% CI: 0.97~2.07 mmHg, *p* < 0.0001), and the WMD between preoperative IOPg and IOP_GAT_ was 1.16 mmHg (95% CI: 0.60~1.73 mmHg, *P* < 0.0001). And the three ∆IOP values from the largest to the lowest were shown as: mean-∆IOPg = 3.83 mmHg, mean-∆IOP_GAT_ = 2.65 mmHg, mean-∆IOPcc = 1.43 mmHg.

**Table 3:** Preoperative IOP and the change of IOP, the unit is mmHg

Name/y	Preoperative			Postoperative			*∆*IOPcc	*∆*IOPg	*∆*IOP_GAT_
	IOPcc	IOPg	IOP_GAT_	IOPcc	IOPg	IOP_GAT_			
Pepose/2007 [18]	15.4 ± 3.2	15.2 ± 3.4	13.8 ± 3.3	13.1 ± 2.0	10.6 ± 2.6	12.0 ± 2.7	2.1 ± 2.6	4.6 ± 2.7	2.6 ± 2.2
Qazi_a/2009 [19]	15.52 ± 3.43	15.72 ± 3.70	14.40 ± 3.27	13.37 ± 2.53	11.29 ± 3.08	11.53 ± 2.45	2.66 ± 3.54	4.41 ± 3.74	4.46 ± 3.68
Denise_d/2011 [20]	15.50 ± 2.50	14.70 ± 2.70	13.40 ± 2.20	15.09 ± 2.30	12.11 ± 2.54	12.40 ± 2.67	0.27 ± 1.91	2.34 ± 2.09	0.95 ± 3.30
Shin/2015 [21]	14.31 ± 2.42	14.19 ± 2.54	13.43 ± 2.19	13.64 ± 2.09	10.27 ± 2.26	10.83 ± 2.83	0.67 ± 2.07	3.92 ± 2.19	2.60 ± 2.51

## Discussion

With the rapid development of corneal refractive surgery technology and the improvement of social living standards, more and more myopic patients choose to undergo the refractive surgery to improve their vision. Meanwhile, the importance of accurately measuring the IOP after corneal refractive surgery for guiding clinical medication and timely discovering secondary diseases is gradually recognized by more and more ophthalmologists [1, 3]. Therefore we conducted a systematic review and meta-analysis on comparison of IOPcc, IOPg and IOP_GAT_ after corneal refractive surgery to gain a more comprehensive conclusion on postoperative IOP.

In the matter of comparison between IOPg and IOP_GAT_ after corneal refractive surgery, our result showed that there was no significant difference between IOPg and IOP_GAT_. That is consistent with most previous studies [3, 17–20, 22]. In addition, almost all studies have suggested that IOP_GAT_ is highly dependent on corneal thickness [17, 21–23], so it is reasonable to believe that IOPg is also associated with corneal thickness. In terms of the comparison between IOPcc and IOP_GAT_ after corneal refractive surgery, our study showed that IOPcc was 2.56 mmHg higher than IOP_GAT_ in general, and the difference between IOPcc and IOP_GAT_ was statistically significant. The relationship between IOPcc and IOP_GAT_ after surgery is also consistent with the existing studies [17–20, 24].

We all know that normal and stable IOP depends on the dynamic balance of volume of ocular contents, rate of aqueous humor production and rate of aqueous humor discharge [25]. After refractive surgery, in spite of the cornea becomes thinner, there is no significant change in the generation and flow of aqueous humor, that is, there have little effect on the aqueous humor circulation, so, the actual IOP will not have a great change theoretically [26]. Based on the assumption that IOP remained basically unchanged before and after refractive surgery, this study also referred to the method of previous researches [3, 27], that was, using the difference of pre- and post-operative IOP (∆IOP) to evaluate which IOP measurements is closer to the real IOP after the surgery. Our study showed that mean-∆IOPg > mean-∆IOP_GAT_ > mean-∆IOPcc, and mean-∆IOP_GAT_ was 1.853 times that of mean-∆IOPcc. So considering the high dependence between IOP_GAT_ and CCT, it is reasonable to speculate that IOPcc may indeed be less dependent on corneal thickness. Previous studies [4, 18, 19] have also mentioned lower percent change in IOPcc measured before and after surgery, and it suggests that IOPcc could partially compensate for the corneal properties of the cornea. Therefore, we inferred that IOPcc may be closer to real IOP after corneal refractive surgery than others.

Although this study was focused on the influence of corneal thickness on IOP after corneal refractive surgery, the measurement value of IOP was actually also affected by mechanical properties [28, 29]. Corneal Visualisation Scheimpflug Technology (Corvis ST; Oculus Optikgerte GmbH, Wetzlar, Germany) is a new noncontact tonometer characterized with high-speed Scheimpflug technology, which facilitated the measurement of IOP. And the biomechanical corrected IOP (bIOP) [30] is purported to be less dependent on biomechanical properties. At present, there are some researches to further explore and verify the IOP measurement of bIOP in healthy people [31], glaucoma [32] and even keratoconus patients [31]. In addition, it has been proposed [33] that bIOP is able to reduce the known correlation between CCT and IOP readings before and after refractive surgery, such as LASIK and small-incision lenticule extraction (SMILE) [33]. However, as Corvis is a newly developed device in recent years, there are few studies on the measurement and comparison of IOP after refractive surgery, so it has not been included in the meta-analysis as ORA and GAT for overall comparison. However, it is believed that with the increase in the number of related literatures, it is also of great significance to conduct meta-analysis of Corvis and other tonometers after refractive surgery.

The choice of the type of corneal refractive surgery is limited by many factors such as the diopter of patient and corneal thickness [34]. Generally, the corrected diopter of corneal lamellar refractive surgery is higher than that of surface refractive surgery. Moreover, the higher the corrected diopter is, the larger the amount of corneal cutting need to take, and the thinner the residual bed thickness of cornea are left [35]. And relatively, the greater the amount of corneal cutting is, the more significant the changes of corneal biomechanical properties will be [29]. IOP measurements affected by CCT and corneal mechanical properties. The stability of biomechanical properties of cornea after the refractive surgery is another possible error source in IOP measurement. In the subgroup analysis of postoperative IOPcc and IOP_GAT_ on surgical procedures, the heterogeneity among the data after corneal surface refractive surgery was zero, while the heterogeneity after corneal lamellar refractive surgery was 87%. Compared with the IOPcc after the corneal lamellar refractive surgery, IOPcc after corneal surface refractive surgery was relatively stable. This result reminds us that the stability of IOPcc after different refractive surgery may be related to the stability of corneal biomechanics. Therefore, it is assumed that the biomechanical properties of cornea after surface refractive surgery may be more stable than those after lamellar surgery. Of course, more clinical data are needed to further substantiate the conclusion.

The use of hormones after corneal refractive surgery is the main influencing factor of steroid-type glaucoma, so monitoring of IOP is particularly important during the postoperative use of hormones [36]. Although the types of hormones used and the time of administration for different surgical methods are not the same, our literature-based research showed that there was no significant correlation between the follow-up time and the size of heterogeneity, regardless of the follow-up time of dividing line for 1 month or 3 months. Considering that there are many factors influencing the occurrence of glaucoma after corneal refractive surgery, the conclusion of this paper needs further verification of clinical cases.

Our reporting also had certain limitations. For instance, we did not consider the effect of sample size on the combined effect size, which might lead to the relatively dominant effect of large sample size data on the results. We also did not conduct meta-regression or subgroup analysis on the age of patients, the main reason was the corneal refractive surgery for patients with a certain age requirements. In general, 18 to 45 was the optimal age for the procedure, so the age of the patients in this study was too concentrated to be grouped again.

## Conclusions

In summary, IOPcc, which is less dependent on CCT, may be more close to the true IOP after corneal refractive surgery compared with IOPg and IOP_GAT_. Moreover, In terms of postoperative recovery of IOP, IOPcc after corneal surface refractive surgery may be more stable than that after lamellar refractive surgery. Further research and validation through more clinical data are needed.

## Data Availability

Data can be shared upon request.

## Abbreviations

bIOP: Biomechanical corrected IOP
CCT: Central corneal thickness
CH: Corneal hysteresis
CI: Confidence interval
CRF: The corneal resistance factor
EPI-LASIK: Epipolis Laser in Situ Keratomileusis
FS-LASIK: Femtosecond Laser-assisted LASIK
GAT: Goldmann applanation tonometer
iCare RBT: iCare rebound tonometer
IOP: Intraocular pressure
IOPcc: Corneal-Compensated Intraocular Pressure
IOPg: Goldmann-correlated Intraocular Pressure
IOP_GAT_: Intraocular pressure measured by GAT
LASEK: Laser-assisted Subepithelial Keratomileusis
LASIK: Laser-assisted in Situ Keratomileusis
NCT: Noncontact tonometer
ORA: Ocular Response Analyzer
PRK: Photorefractive Keratectomy
SMILE: Small-incision lenticule extraction
WMD: Weighted mean difference
